# A Reaction-Diffusion Model of ROS-Induced ROS Release in a Mitochondrial Network

**DOI:** 10.1371/journal.pcbi.1000657

**Published:** 2010-01-29

**Authors:** Lufang Zhou, Miguel A. Aon, Tabish Almas, Sonia Cortassa, Raimond L. Winslow, Brian O'Rourke

**Affiliations:** 1 Division of Cardiology, Department of Medicine, The Johns Hopkins University School of Medicine, Baltimore, Maryland, United States of America; 2 Department of Biomedical Engineering, The Johns Hopkins University School of Medicine, Baltimore, Maryland, United States of America; Medical College of Wisconsin, United States of America

## Abstract

Loss of mitochondrial function is a fundamental determinant of cell injury and death. In heart cells under metabolic stress, we have previously described how the abrupt collapse or oscillation of the mitochondrial energy state is synchronized across the mitochondrial network by local interactions dependent upon reactive oxygen species (ROS). Here, we develop a mathematical model of ROS-induced ROS release (RIRR) based on reaction-diffusion (RD-RIRR) in one- and two-dimensional mitochondrial networks. The nodes of the RD-RIRR network are comprised of models of individual mitochondria that include a mechanism of ROS-dependent oscillation based on the interplay between ROS production, transport, and scavenging; and incorporating the tricarboxylic acid (TCA) cycle, oxidative phosphorylation, and Ca^2+^ handling. Local mitochondrial interaction is mediated by superoxide (O_2_^.−^) diffusion and the O_2_^.−^-dependent activation of an inner membrane anion channel (IMAC). In a 2D network composed of 500 mitochondria, model simulations reveal ΔΨ_m_ depolarization waves similar to those observed when isolated guinea pig cardiomyocytes are subjected to a localized laser-flash or antioxidant depletion. The sensitivity of the propagation rate of the depolarization wave to *O_2_^.−^* diffusion, production, and scavenging in the reaction-diffusion model is similar to that observed experimentally. In addition, we present novel experimental evidence, obtained in permeabilized cardiomyocytes, confirming that ΔΨ_m_ depolarization is mediated specifically by O_2_^.−^. The present work demonstrates that the observed emergent macroscopic properties of the mitochondrial network can be reproduced in a reaction-diffusion model of RIRR. Moreover, the findings have uncovered a novel aspect of the synchronization mechanism, which is that clusters of mitochondria that are oscillating can entrain mitochondria that would otherwise display stable dynamics. The work identifies the fundamental mechanisms leading from the failure of individual organelles to the whole cell, thus it has important implications for understanding cell death during the progression of heart disease.

## Author Summary

Cardiac cell injury and death is a key component of cardiac diseases such as heart failure or myocardial infarction, thus it is important to understand the earliest steps leading up to irreversible cell damage. Mitochondria are the organelles responsible for generating the energy required to keep the cell running, yet they are particularly vulnerable to damage by toxic byproducts of metabolism, which include reactive oxygen species (ROS). ROS wreak havoc on cellular functions by attacking proteins, lipids and DNA, so the cardiac cell has evolved sophisticated defenses to remove them. The work we present in this paper using a computer model of the mitochondrial network describes how ROS generated inside the cell can spread from one mitochondrion to the next in a positive feedback process known as ROS-induced ROS release. Understanding this process will help us to find ways to intervene in this catastrophic mechanism to prevent loss of cell function and the associated cardiac arrhythmias and contractile problems leading to sudden cardiac death.

## Introduction

The spatial and temporal organization of the mitochondrial network is crucial for understanding its function in cells [1],[2]. Complex spatiotemporal factors not only contribute to physiological signaling [3]–[6], but determine the fate of the cell under stress. In heart cells, we have previously studied how the lattice-like packing arrangement of the mitochondrial network lends itself to propagation of bioenergetic signals [7] and to synchronization of self-organized oscillations in ROS, redox potential, and mitochondrial inner membrane potential (ΔΨ_m_) in response to pathological stimuli [8]–[10]. Moreover, the scaling of instability of the mitochondrial network to the whole-cell and whole-organ levels has been shown to underlie the electrophysiological and contractile dysfunction associated with cardiac disease [11]–[13].

Only recently have we begun to understand the mechanisms responsible for stable, unstable, and oscillatory modes of mitochondrial bioenergetics, by combining experiments with mathematical model development. In studies of isolated cardiomyocytes subjected to localized oxidative stress, we elucidated the role of ROS in triggering autonomous synchronized oscillations of mitochondrial energetics [8],[9]. An emergent low frequency (0.01Hz), high amplitude, limit cycle oscillation of ΔΨ_m_ was observed that spanned the length and breadth of the cardiomyocyte, but only after a considerable delay following the initial perturbation (local laser flash). We found that the abrupt transition in ΔΨ_m_ was preceded by the gradual increase of oxidative stress in the network, more specifically, when ∼60% of the mitochondria accumulated ROS to a threshold level. We referred to the state of the mitochondrial network just before depolarization as the point of “mitochondrial criticality” [9]. In the critical state, a small perturbation anywhere in the network can lead to the propagation of a ΔΨ_m_ depolarization wave. The mechanism of this phenomenon involves a mitochondrial ROS-induced ROS release mechanism (a term originally coined by Zorov et al [14],[15]), triggering an energy dissipating inner membrane anion channel (IMAC) that is inhibited by mitochondrial benzodiazepine receptor (mBZR) ligands, but not by inhibitors of the permeability transition pore [8]–[10].

A computational model of a ROS-dependent single mitochondrion oscillator was constructed to study the dynamics of the system [16] and it could reproduce the main features observed experimentally, including bursts of ROS release to the extramitochondrial space during rapid ΔΨ_m_ depolarization. Interestingly, in addition to the slow, large amplitude oscillation mode most closely associated with the pathological state, the model displayed a wide range of frequencies and amplitudes, suggesting the possibility that mitochondrial ROS release may be operating under physiological conditions as well. A subsequent study provided experimental evidence to support the idea that the mitochondrial network is organized as a collection of weakly coupled oscillators under physiological conditions that couple more strongly under stress to produce slow, synchronized waves and oscillations [3].

Because the initial collapse of the mitochondrial network initiates a cascade of events including activation of sarcolemmal K_ATP_ channels, alteration of the electrical excitability of the cardiomyocyte, and ultimately cardiac arrhythmias [11],[17], it is important to gain a precise understanding of the mechanisms responsible for signal propagation between mitochondria in the network, in order to find ways to interrupt potentially catastrophic events. Thus, in the present work, we test the proposal that *O_2_^.−^* diffusion from one mitochondrion to its nearest neighbors is responsible for the propagation of ΔΨ_m_ depolarization waves and synchronization of oscillation in the mitochondrial network by building one dimensional (1D) and two dimensional (2D) reaction-diffusion models of the mitochondrial network. We show that model simulations closely resemble ΔΨ_m_ depolarization wave propagation observed in experiments in response to oxidative stress, and remarkably, we find that a few oscillating mitochondria can entrain the entire network into an oscillatory mode even if the majority of the mitochondrial lattice is not in the oscillatory parametric domain. We propose that perturbing a minimal number of mitochondria is sufficient to trigger cell-wide responses through ROS-dependent coupling of mitochondria in the network.

## Results

**Figure 1:**
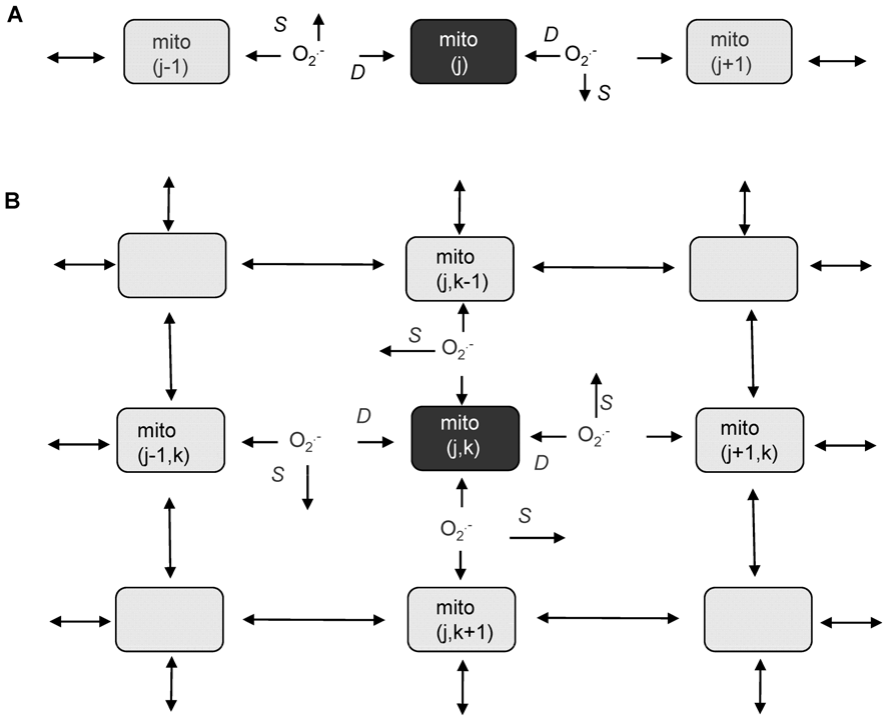
Schemes of the RD-RIRR mitochondrial network model. In the RD-RIRR model (A: 1D and B: 2D), neighboring mitochondria are chemically coupled with each other through *O_2_^.−^* diffusion. Light and dark gray indicate polarized and depolarized mitochondria, respectively. Arrows indicate release of superoxide anion, O_2_^.−^, and its effect on mitochondrial neighbors. *D* stands for O_2_^.−^_i_ diffusion, and *S* for O_2_^.−^_i_ scavenging by Cu,Zn SOD and catalase.

We first simulated the propagation of a wave of ΔΨ_m_ depolarization, as observed in experiments performed with isolated guinea pig ventricular myocytes subjected to a localized laser flash [8]. In order to determine if *O_2_^.−^* diffusion and the RIRR mechanism is able to account for transmission of the wave, we started with the 1D version of the RD-RIRR network model (Fig. 1A), with the individual mitochondrial nodes either in the stable or oscillatory parametric domains. The 1D model was employed as a proof of principle, to assess under which conditions a wave of depolarization could be initiated from a stimulus consisting of a mitochondrion generating enough *O_2_^.−^* to reach the immediate neighbors at supra-threshold levels. This local *O_2_^.−^* diffusion thus elicits the opening of IMAC and the depolarization of ΔΨ_m_ in the neighboring mitochondrion.

### 

#### Propagation of ΔΨ_m_ depolarization in 1D mitochondrial network

**Figure 2:**
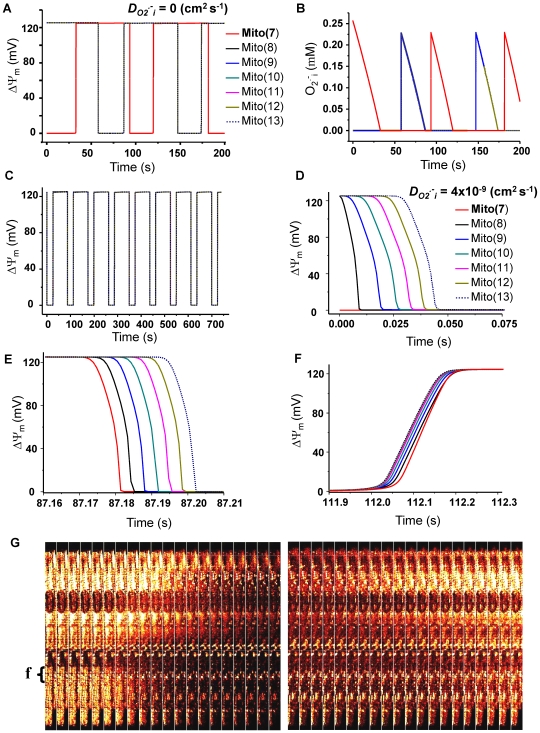
Spatial propagation of ΔΨ_m_ depolarization in the oscillating mitochondrial network: computer simulations using the 1D RD-RIRR model and experimental evidence. A network consisting of a linear array of 13 mitochondria was analyzed. The mitochondrion in the center (mito_7) was initially depolarized whereas the others on both sides were polarized. A and B) The dynamics of ΔΨ_m_ and O_2_^.−^_i_ without *O_2_^.−^* coupling through diffusion between mitochondria (i.e., *D*_O2_^.−^_i_ = 0). Mitochondria in the row are differentiated by colors, and only one side of the row is shown (i.e., from mito_7 to mito_13). C) ΔΨ_m_ dynamics in the presence of O_2_^.−^_i_ diffusion (*D*_O2_^.−^_i_ = 4×10^−9^ cm^2^ s^−1^). D and E) Expanded records of the first and second depolarization waves propagating from mito_7 (depolarized in D) through mito_8 (red line) until mito_13. F) Expanded record of the second repolarization wave. G) Montage of the experimental evidence of ΔΨ_m_ depolarization (left) and repolarization (right) wave propagation obtained in a single cardiomyocyte during laser flash-induced whole cell mitochondrial oscillations. Notice that the mitochondria in the network that depolarized last were the first to repolarize. Mitochondrial oscillations were triggered with a laser flash in cardiomyocytes labeled with 100 nM TMRE. Frame acquisition was every 500 msec. Other imaging conditions were as described in Materials and Methods and [9]. The main parameters changed in the simulations in order to obtain mitochondrial oscillations were the *shunt* ( = 0.14), and *etSOD* ( = 1.45 µM) whereas all other parameters were as described in [16] and also listed in the Supplemental Materials (Table S2).

**Figure 3:**
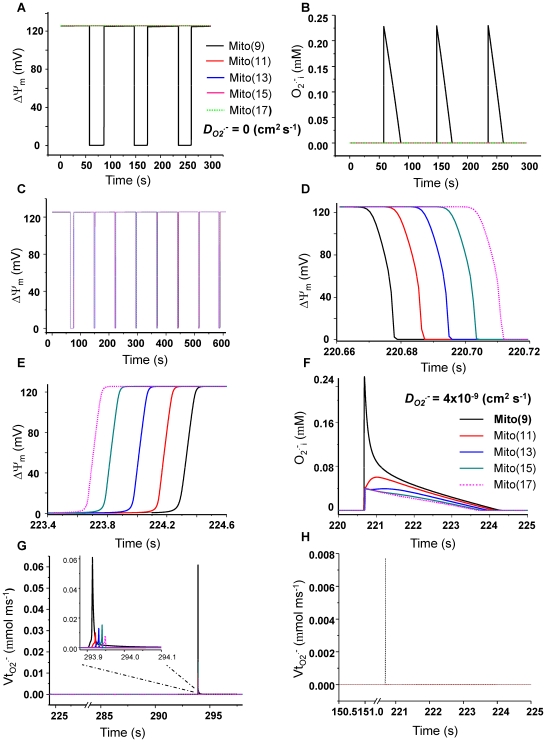
Spatial propagation of ΔΨ_m_ depolarization in non-oscillating mitochondrial networks using the 1D RD-RIRR model. A network consisting of a row of 17 mitochondria was analyzed. The mitochondrion in the center (mito_9) was initialized with parameters within the oscillatory domain (*shunt* = 0.14) whereas the other mitochondria were within the nonoscillatory stable range of parameters (*shunt* = 0.02). *etSOD* was 1.45 µM for all mitochondria in the network. A and B) The dynamics of ΔΨ_m_ and O_2_^.−^_i_ without *O_2_^.−^* coupling through diffusion between mitochondria (i.e., *D*_O2_^.−^_i_ = 0). Mitochondria in the row are differentiated by colors, and only part of one side of the row is shown (i.e., from mito_9 to mito_17). C) ΔΨ_m_ dynamics in the presence of *O_2_^.−^* diffusion (*D*_O2_^.−^_i_ = 4×10^−9^ cm^2^ s^−1^). D and E) Expanded records of the third depolarization (D) and repolarization (E) waves spanning mito_9 through mito_17. F) Expanded record of *O_2_^.−^* during a repolarization wave showing the dynamics of O_2_^.−^_i_ in each mitochondrion (color coded) with mito_9 showing the highest amount of cytoplasmic O_2_^.−^. G and H) The rate of *O_2_^.−^* release from mitochondria to the periplasmic space (*Vt*_O2_^.−^_i_) in the network (G) or in an isolated mitochondrion (H). All other parameters were as described in [16] and also listed in the Supplemental Materials (Table S2).

Two different sets of initial parametric conditions were chosen for the *in silico* experiments shown (Fig. 2A–2F and Fig. 3). In the first simulation (Fig. 2), both the mitochondrion at the center of the row, as well as the other mitochondria in the array (13 element array), had parameters within the oscillatory domain, whereas in the second simulation (Fig. 3), only the central mitochondrion (mito_9) was in the oscillatory domain (shunt = 0.14) but the others were not (shunt = 0.02).

With all the mitochondria in the same parametric space (oscillatory domain), the model was initialized with conditions set such that the central mitochondrion (mito_7) started in a depolarized state (i.e., low ΔΨ_m_ and high *O_2_^.−^* concentration) while the others were still polarized. In the absence of *O_2_^.−^* diffusion (O_2_^.−^_i_ diffusion coefficient, *D*_O2_^.−^ *_i_* = 0), all mitochondria oscillated independently (Fig. 2A and 2B). In this case, the initially depolarized mito_7 repolarized at 32.2 s and then continued with limit cycle oscillations. Mitochondria that were polarized at time zero (i.e., mito_1 to 6 and mito_8 to 13) depolarized at 57.8 s and then oscillated independently of their neighbors. All mitochondria displayed the same oscillation period (87 s) as reflected in the changes in membrane potential (Fig. 2A), the cytoplasmic *O_2_^.−^* (O_2_^.−^_i_) concentration (Fig. 2B), mitochondrial *O_2_^.−^* (O_2_^.−^_m_), or NADH (not shown).

With the same parameter settings, the simulation was repeated in the presence of O_2_^.−^_i_ diffusion (*D*_O2_^.−^ *_i_* = 4×10^−9^ cm^2^ s^−1^), which revealed a significantly different pattern of ΔΨ_m_ (Fig. 2C) and O_2_^.−^_i_ oscillations, consistent with propagation of the depolarization (Fig. 2D) and O_2_^.−^. Upon depolarization of the central mitochondrion (mito_7), there was a short lag (∼10 ms), and then mitochondria 8 through 13 depolarized sequentially (Fig. 2D). ΔΨ_m_ depolarization propagated identically in both directions (e.g., from mito_6 to mito_1; results not shown). Notably, both in the model simulation (Fig. 2E and 2F, see also Fig. 3 below) and in experiments performed in isolated cardiomyocytes (Fig. 2G), the mitochondrion that depolarized first was the last to repolarize.

**Figure 4:**
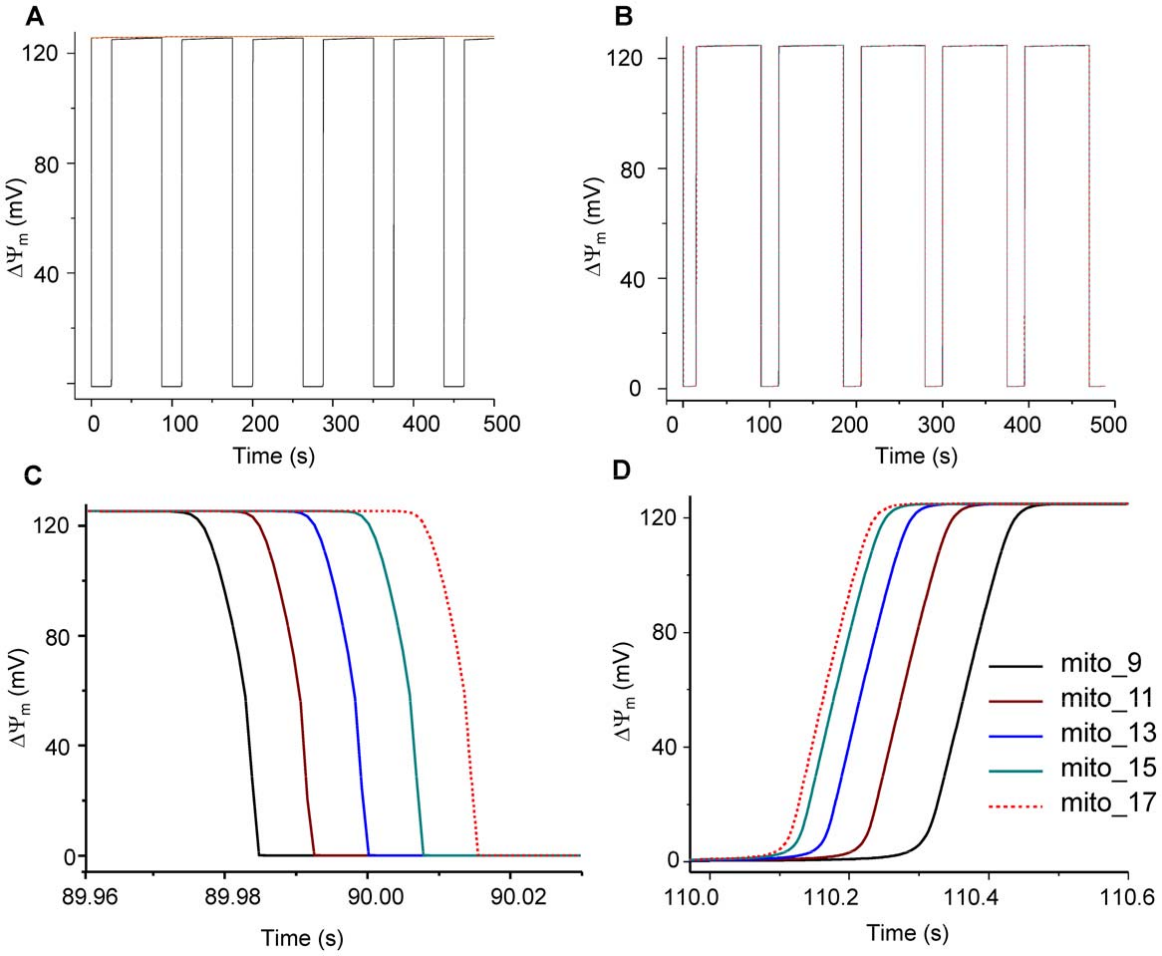
Simulation of the effect of increasing the ROS shunt in one mitochondrion on row oscillatory behavior using the 1D model. (A) represents ΔΨ_m_ without the presence of ROS diffusion (*D*_O2_^.−^_i_ = 0); (B) represents ΔΨ_m_ with the presence of ROS diffusion (*D*_O2_^.−^_i_ = 4×10^−9^ cm^2^ s^−1^); (C) enlarged ΔΨ_m_ depolarization wave; (D) enlarged ΔΨ_m_ depolarization wave; *etSOD* = 1.43×10^−3^ mM and all other parameters are as listed in Supplemental Materials Tables S2.1, S2.2, S2.3, S2.4.

In a second set of simulations, we investigated whether oscillation in a single mitochondrion could entrain the rest of the network, even though the target mitochondria were initialized with parameters outside the oscillatory regime. The network consisted of a linear array of 17 mitochondria in which the mitochondrion in the center (mito_9) was initialized with an enhanced rate of *O_2_^.−^* production by increasing the *shunt* parameter (defined as the fraction of respiration diverted to *O_2_^.−^* production) from its baseline value of 0.02 (2%) to 0.14 (14%). As shown above (Fig. 2), in the absence of O_2_^.−^_i_ diffusion, mitochondria behave independently and only mito_9 oscillated (Fig. 3A and 3B). Coupling mitochondria through O_2_^.−^_i_ diffusion (*D*_O2_^.−^ *_i_* = 4×10^−9^ cm^2^ s^−1^) resulted in entrainment of the oscillatory mode, with mito_9 triggering a depolarization wave which propagates from the center to the ends of the array (mito_17; Fig. 3D and mito_1; not shown). In agreement with experimental results reported previously [9], repolarization begins from the mitochondrion that depolarized last, that is, it proceeds from mito_17 or mito_1 to the center of the row (Fig. 3E). Similar results were obtained by simulating a reduction in antioxidant capacity by decreasing *etSOD* (the Cu, Zn SOD concentration) for mito_9 from 2×10^−3^ to 1.43×10^−3^ mM (Fig. 4).

Entrainment of mitochondrial oscillation in the network depended on the local *O_2_^.−^* increase introduced by the perturbation of the mitochondrion in the center of the row (mito_9), which generated periplasmic *O_2_^.−^* concentrations (O_2_^.−^_i_) that were much higher than the neighboring mitochondria (Fig. 3F), as reflected by the high transport rate of O_2_^.−^_i_ (*Vt*_O2_^.−^_i_) from the matrix to the cytoplasm in mito_9 (Fig. 3G). Enhancement of the local O_2_^.−^_i_ release in mitochondria far from the central mitochondrion depended on ROS diffusion. With a *D*_O2_^.−^_i_ of 4×10^−9^ cm^2^ s^−1^ (Fig. 3F), propagation of a O_2_^.−^_i_ wave and spikes in *Vt*_O2_^.−^_i_ (Fig. 3G) could be demonstrated in mitochondria at the ends of the array, but not when diffusional coupling was eliminated (Fig. 3H). Furthermore, in the absence of O_2_^.−^_i_ diffusion, the distant mitochondria remained polarized and their local *O_2_^.−^* levels changed negligibly (Fig. 3A and 3B).

#### Superoxide as the trigger of ΔΨ_m_ depolarization

**Figure 5:**
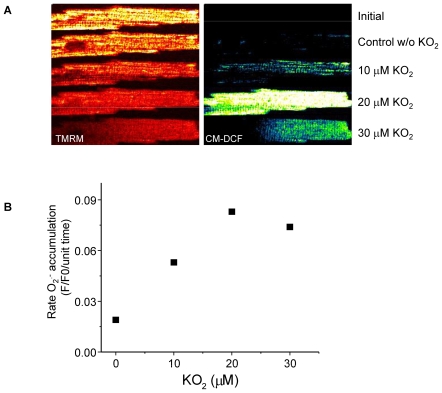
Mitochondrial *O_2_^.−^* and ΔΨ_m_ in response to increased exogenous *O_2_^.−^* in saponin-permeabilized cardiomyocytes. Myocytes were loaded with TMRM (100 nM) and CM-H_2_DCFDA (2 µM) for at least 20min and imaged using two-photon laser scanning fluorescence microscopy (see Materials and Methods). After loading, the excess dye was washed out and the cells were briefly superfused with a permeabilizing solution (saponin) as previously described [10]. After permeabilization, the myocytes were continuously perfused with an intracellular solution containing GSH∶GSSG at a ratio of 300∶1. The TMRM was included in the medium to avoid depletion of the probe during depolarization-repolarization cycles. A) The TMRM and CM-DCF images of a permeabilized cardiomyocyte at time zero after loading and before (top row image) or after permeabilization and 5min imaging under control conditions (Control, second row) or the presence of KO_2_ (10 µM, third row; 20 µM, fourth row; 30 µM, fifth row) after 3min equilibration in each case. RIRR-mediated ΔΨ_m_ depolarization without a permeability transition occurs at the two lower concentrations, while loss of the CM-DCF probe (∼500MW) from the mitochondrial matrix due to PTP opening occurs at 30 µM KO_2_. B) The rates of *O_2_^.−^* accumulation as a function of KO_2_ concentration. Slopes were calculated when the linear rate of change of the CM-DCF signal stabilized under each condition.

**Figure 6:**
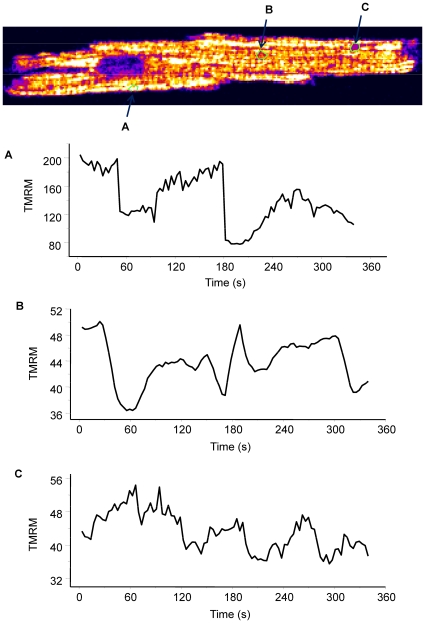
Plot of membrane potential of selected mitochondrial groups that display limited cycle oscillations. Guinea-pig ventricular myocyte was loaded with TMRM and CM-DCF and then partially permeabilized with 25µg ml^−1^ saponin as described in Materials and Methods. Image frames were collected every 3.5 seconds using a two-photon laser scanning fluorescence microscope. See also the supplemental materials Video S1.

The original ROS-dependent mitochondrial oscillator model [16], which was based on experimental observations, considered cytoplasmic *O_2_^.−^* as the primary free radical species that would increase in IMAC open probability in an autocatalytic process. H_2_O_2_ was ruled out because superoxide dismutase mimetics, which should enhance H_2_O_2_ accumulation, suppressed the oscillations in ΔΨ_m_ [8]. To test the assumption that *O_2_^.−^* could directly trigger IMAC opening we applied increasing concentrations of KO_2_ to partially permeabilized myocytes. Increasing the exogenous cytoplasmic KO_2_ concentration, as a source of O_2_^.−^, from 10 to 20 µM, elicited progressive ΔΨ_m_ depolarization and increased the rate of mitochondrial *O_2_^.−^* accumulation (Fig. 5A and 5B). Reversible ΔΨ_m_ depolarizations were observed at intermediate concentrations of KO_2_ (10 to 20 µM, Video S1 and Fig. 6). However, exposure of the cell to 30 µM KO_2_ induced an irreversible collapse of ΔΨ_m_, accompanied by the complete release of the *O_2_^.−^* sensor indicative of permeability transition pore (PTP) opening (Fig. 5A, bottom row; see also Supplemental Materials Video S2).

#### Propagation of ΔΨ_m_ depolarization and *O_2_^.−^* in the 2D RD-RIRR mitochondrial network model

**Figure 7:**
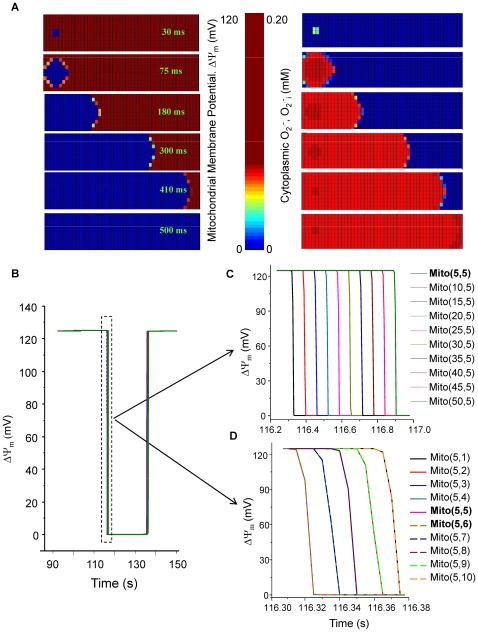
Simulation of ΔΨ_m_ depolarization wave propagation in the 2D RD-RIRR model. In a network consisting of 500 mitochondria (10×50), 6 of which were initially depolarized while the others stayed polarized. A) Spatial propagation of ΔΨ_m_ depolarization (left) and O_2_^.−^_i_ (right) with a rate of 200 µm s^−1^; B) ΔΨ_m_ dynamics, and (C, D) expanded views of ΔΨ_m_ depolarization in the x and y directions, respectively, corresponding to the 500 mitochondria in the network. *Shunt* = 0.14; *etSOD* = 1.43 µM; *D*_O2_^.−^_i_ = 2×10^−10^ cm^2^ s^−1^. All other parameters were as described in [16] and also listed in the Supplemental Materials (Table S2).

The propagation of RIRR was also demonstrated in the 2D mitochondrial network model, which consisted of five hundred (10×50) mitochondria. The parameters of the whole array were initialized to represent a condition of high oxidative stress by increasing the *shunt* parameter to 0.14 to simulate a mitochondrial network at criticality. Approximately 1% (6 out of 500) of the mitochondrial network was induced to undergo depolarization. A local increase in O_2_^.−^_i_ concentration and depolarization of ΔΨ_m_ in this area is evident, similar to previous experiments in which we applied a localized laser flash to a fraction of the mitochondrial network [8] (but see discussion for the limitations of this analogy). ΔΨ_m_ depolarization propagated outward in all directions from the six perturbed mitochondria and then appeared as a longitudinal wave as the edges of the array were encountered (Fig. 7A, left montage). A wave of increased O_2_^.−^_i_ accompanied the ΔΨ_m_ depolarization wave (Fig. 7A, right montage). The wave propagation rates in the *x* (Fig. 7C) and *y* (Fig. 7D) direction are also illustrated as a sequential ΔΨ_m_ plots.

**Figure 8:**
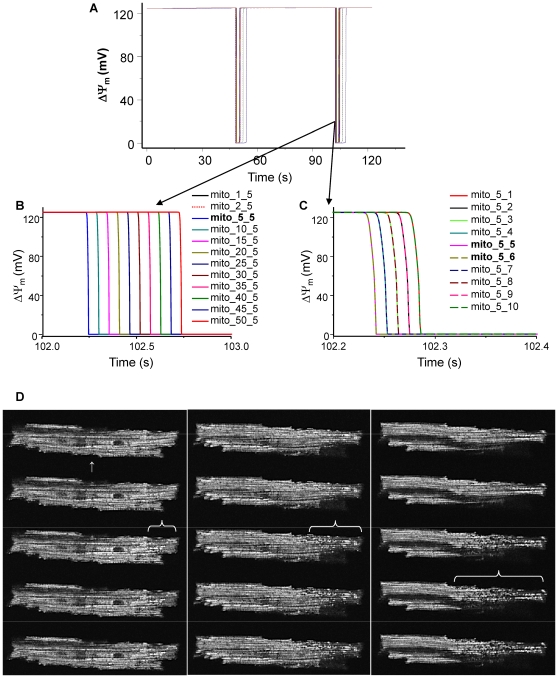
Increasing the fraction of *O_2_^.−^* production from 0.02 to 0.14 in ∼1% of 500 mitochondria induces propagation of membrane potential depolarization wave and elicits whole network oscillations. A) ΔΨ_m_; B) enlarged ΔΨ_m_ depolarization wave in the x direction; and (C) enlarged ΔΨ_m_ depolarization wave in the y direction. For mito(j,k) (j = 4 or 5; k = 5 or 6), shunt = 0.14; for all other mitochondria, shunt = 0.02. Other parameters and initial conditions were same for all mitochondria and listed in Supplemental Materials Tables S2.1, S2.2, S2.3, S2.4. *D_O2_^.−^_i_* = 4×10^−11^ cm^2^ s^−1^. D) Selected images of a pair of TMRM-loaded canine ventricular myocytes (cell-cell junction indicated by the white arrow in left panel) which displayed 22 consecutive oscillations (see time series in Supplemental Materials, Video S3) that originated at the end of the righthand cell. Images within each panel were acquired at a 1 second frame rate and depict the 1st (left panel), 11th (center panel), and 14th cycle of ΔΨ_m_ oscillation (right panel). Entrainment was indicated by an increase in the area of the oscillating cluster after a number of cycles until the whole cell (up to the border of the neighboring cell) was included.

We next investigated whether a local (6 out of 500) increase in O_2_^.−^_i_ could spread to the rest of the mitochondria in the 2D RD-RIRR model even though the initialization parameters in the majority of the network were outside of the oscillatory domain. Similar to the results of the 1D simulation, we observed that oscillation in just 1% of the 2D network was sufficient to entrain the whole system (Fig. 8A–C). All mitochondria had the same initial and parametric conditions (*shunt* = 0.02) except for the 6 triggering mitochondria (*shunt* = 0.14). *D*_O2_^.−^_i_ was 4×10^−9^ cm^2^ s^−1^ for the entire network. The ability of an oscillating region of the network to entrain a previously quiescent region was also demonstrated experimentally, as evidenced by the gradually increasing area of synchronous oscillation with multiple cycles of ΔΨ_m_ depolarization (Fig. 8D; canine ventricular myocyte, see also Supplemental Materials Video S3).

**Figure 9:**
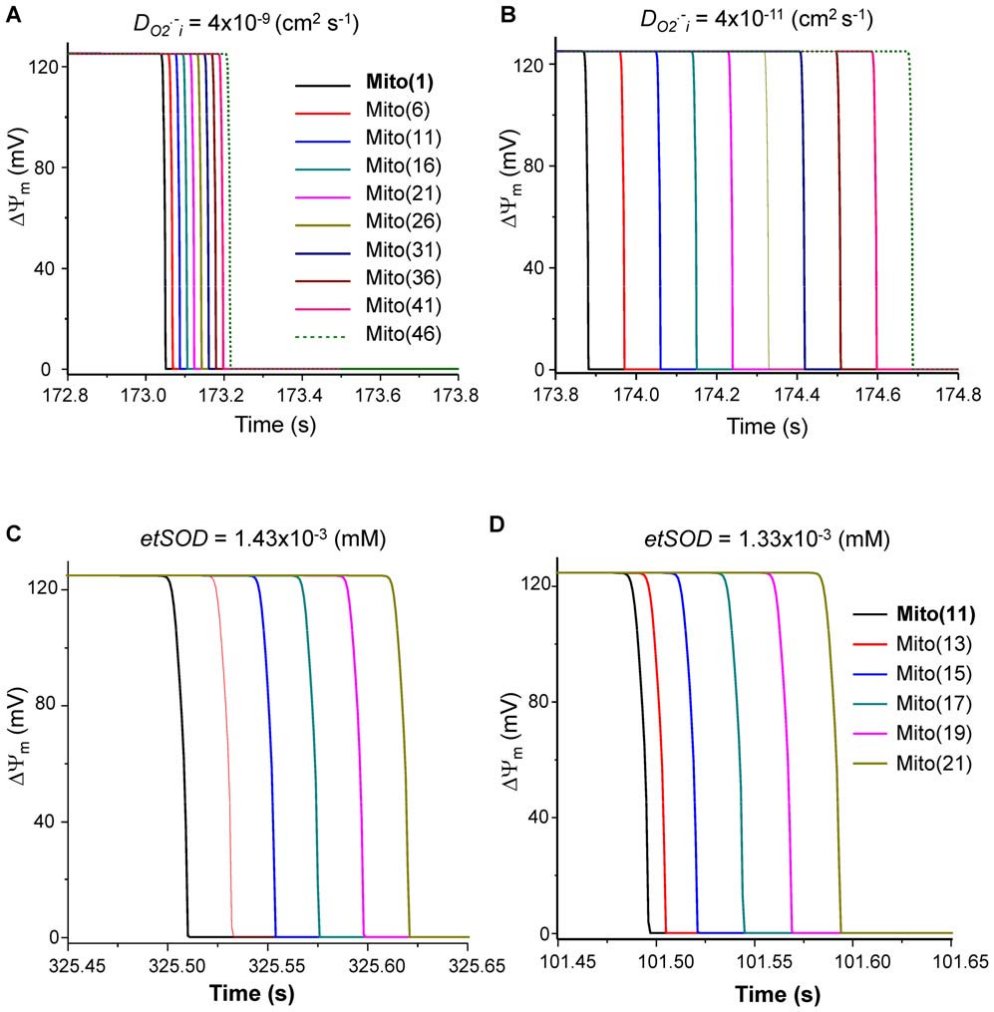
Effect of *D*_O2_^.−^_i_ or *etSOD* on the propagation rate of ΔΨ_m_ depolarization wave. A and B) Increasing *D*_O2_^.−^_i_ by two orders of magnitude (from 4×10^−11^ to 4×10^−9^ cm^2^ s^−1^) accelerated wave propagation from 155 to 486 µm s^−1^. C and D) decreasing etSOD from 1.43 to 1.33 mM decreased the propagation time from 126 to 110 ms. *shunt* = 0.14, *etSOD* = 1.43 µM and *D*_O2_^.−^_i_ = 4×10^−9^ cm^2^ s^−1^ except where specifically indicated. Other parameters and initial conditions were the same for all mitochondria (Supplemental Materials, Tables S2 and S3).

Figure 9 shows the effect of varying the parameters *D*_O2_^.−^_i_ or *etSOD* on the rate of propagation of the ΔΨ_m_ depolarization wave. Decreasing *D*_O2_^.−^_i_ from 4×10^−9^ to 4×10^−11^ cm^2^ s^−1^ significantly decreased the speed of ΔΨ_m_ depolarization (from 486 to 155 µm s^−1^, Fig. 9A and 9B). A ΔΨ_m_ depolarization wave propagation rate of 26 µm s^−1^, similar to that observed experimentally ( = 22 µm s^−1^; [9]) was obtained with *D*_O2_^.−^_i_ = 4×10^−14^ cm^2^ s^−1^. Similarly, ΔΨ_m_ repolarization was slowed when *D*_O2_^.−^_i_ was reduced (data not shown). The rate of wave propagation was also affected by the O_2_^.−^_i_ scavenging capacity: when etSOD was decreased from 1.43×10^−3^ to 1.33×10^−3^ mM, the propagation time (for 10 mitochondria) decreased from 126 ms to 110 ms (Fig. 9C and 9D). Taken together, these results indicate that local gradients of cytoplasmic O_2_^.−^_i_, determined by diffusion and scavenger capacity, play a significant role in determining the rate of propagation of the ΔΨ_m_ depolarization and repolarization waves.

## Discussion

The present results demonstrate that the observed emergent macroscopic properties of the mitochondrial network can be reproduced in a reaction-diffusion model of RIRR. Moreover, the simulations have uncovered a novel aspect of the synchronization mechanism, which is that clusters of mitochondria that are in the oscillatory domain of the parametric space can entrain mitochondria that would otherwise display stable dynamics.

Propagation of electrical and Ca^2+^ signals within and between cells in the heart is essential for cardiac function, but less is understood about propagation of signals between organelles [18]. Several hypothetical mechanisms have been previously suggested to explain how ΔΨ_m_ depolarization may spread throughout the mitochondrial network of the cardiac myocyte. Amchenkova et al [19] proposed that there may be direct electrical continuity between mitochondria; however, more recent data showing that individual mitochondria or groups of mitochondria can be depolarized with little effect on their immediate neighbors argues against direct coupling. Ichas et al [20] provided evidence for propagated mitochondrial Ca^2+^-induced Ca^2+^ release mediated ΔΨ_m_ depolarization that involved the activation of the PTP. A mitochondrial ROS-induced ROS release mechanism was described by Zorov *et al* [14] to explain how laser-induced depolarization generates a large burst of *O_2_^.−^* generation from the electron transport chain upon activation of the PTP, which can occur independently of Ca^2+^.

In previous work we investigated whether or not the PTP and/or Ca^2+^ was involved in the mechanism of whole-cell ΔΨ_m_ oscillation ([8]; see [21] for a review). Briefly, several lines of evidence ruled out both the PTP and Ca^2+^ as playing a role in the mitochondrial oscillations observed in heart cells. With regard to Ca^2+^: *(i)* the myocytes were studied under quiescent, minimally Ca^2+^-loaded conditions, and no sarcomere shortening was evident; *(ii)* inhibition of the sarcoplasmic reticulum Ca^2+^ pump or mitochondrial Ca^2+^ handling did not influence flash-induced mitochondrial oscillations; *(iii)* extensive buffering of intracellular Ca^2+^ with 1 mM EGTA did not affect flash-induced oscillations. The possible contribution of the PTP was ruled out by the following evidence: *(i)* cyclosporin A did not block the transitions, and *(ii)* small (600 MW) fluorophores were not lost from the mitochondrial matrix upon depolarization. More recently, we have provided extensive evidence showing that IMAC and PTP open sequentially as a function of the glutathione redox status in permeabilized cardiomyocytes [22].

Studies from our laboratory demonstrated that in cardiomyocytes, mitochondrial ΔΨ_m_ depolarization and redox potential during metabolic stress can be highly synchronized throughout the mitochondrial network, displaying complex behaviors including wave propagation within and between cells [7] and limit cycle oscillations [7],[8],[16]. Using highly localized laser excitation of less than 1% of the cellular volume to induce *O_2_^.−^* release and ΔΨ_m_ depolarization, we showed that after several minutes of delay, spatiotemporally synchronized oscillations in ΔΨ_m_, O_2_^.−^, NADH, and GSH [8],[9],[16], spanning the entire mitochondrial network of the cardiomyocyte, can occur. This phenomenon could be prevented or acutely reversed by interrupting mitochondrial *O_2_^.−^* generation, increasing antioxidant capacity, or blocking IMAC, and neither Ca^2+^ nor PTP opening were involved in this response [8]. These responses could be described by a computational model involving RIRR and the activation of IMAC [16].

Employing the RD-RIRR mitochondrial network model, the present work successfully reproduces several experimental observations, including: i) ΔΨ_m_ redox wave propagation and its spatial-dependence on *O_2_^.−^* diffusion, production, and scavenging, and ii) synchronization of independent mitochondrial oscillators. Interestingly, a new feature was revealed by the 2D model simulation - *entrainment* of mitochondrial ΔΨ_m_ oscillation in mitochondria that would otherwise show stable behavior and low *O_2_^.−^* production. Finally, another important achievement was the direct confirmation of a key component of the oscillatory mechanism and model, which was the experimental demonstration that *O_2_^.−^* itself can induce ΔΨ_m_ depolarization and mitochondrial *O_2_^.−^* accumulation in permeabilized cardiomyocytes. The role of *O_2_^.−^* was previously inferred from the effects of superoxide dismutase mimetic compounds [8].

Reaction-diffusion theory (pioneered by Turing [23]), as a basis for pattern formation in biological or chemical systems, emphasizes the importance of two components; an autocatalytic reaction producing a local product (mediator), and the transport of this product by diffusion away from the source. This process can give rise to spontaneous symmetry-breaking and the appearance of self-organized spatial patterns including waves and oscillations [24]–[30]. With respect to the present model, the reaction consists of the reduction of O_2_ to produce ROS (specifically O_2_^.−^) driven by mitochondrial electron donors (e.g., NADH). The local concentration of *O_2_^.−^* around the mitochondrion is shaped by several others factors, including buffering by the antioxidant reactions and transport of *O_2_^.−^* across the mitochondrial membrane. As in many reaction-diffusion systems, the mitochondrial oscillator also displays an inhibitory, or a self-limiting, mechanism; the concentration of *O_2_^.−^* at the activator site on IMAC decreases during ΔΨ_m_ depolarization because *i)* the rate of scavenging by SOD increases as *O_2_^.−^* accumulates and *ii)* the driving force for *O_2_^.−^* production and transport diminishes. Diffusion of the *O_2_^.−^* to neighboring mitochondria is shaped by the *O_2_^.−^* diffusion coefficient (*D*_O2_^.−^ *_i_*) and the amount of the *O_2_^.−^* scavenger enzyme, *etSOD*, which consequently determines the rate of propagation of ΔΨ_m_ depolarization through the network. As expected, increasing *etSOD* slowed down the depolarization wave. The rate of propagation of the depolarization wave in the model corresponded to 26 µm s^−1^ with low *D*_O2_^.−^ *_i_* (on the order of 10^−14^), which compares well with the experimentally determined 22 µm s^−1^ [9] at 37°C. A restricted diffusion range of *O_2_^.−^* in cells is consistent with experimental data; however, the actual diffusion coefficient of *O_2_^.−^* in cells (with antioxidant systems disabled) has not been determined and is likely to be influenced by local reactions with other molecules and molecular crowding around mitochondria, which would decrease the effective volume and increase the viscosity of the medium. This assumption of restricted diffusion is represented by the low *D*_O2_^.−^ *_i_* in the model.

Scaling of local interactions in complex dynamic systems to produce global emergent behavior is common in physical, social, financial and biological networks [31]. The RD-RIRR model illustrates how local neighbor-neighbor interactions (1–2 µm distance) can lead to long distance spatiotemporal patterns. Mechanistically, in cell-wide mitochondrial oscillations, propagation is mediated by regenerative RIRR between neighboring mitochondria. Our previous work treated the network as a percolation lattice [9] and we postulated that near the percolation threshold of the system, any mitochondrion within the spanning cluster (i.e., one which is close to the critical level of ROS accumulation in the matrix) can depolarize, producing a burst of *O_2_^.−^*, which diffuses to its neighbor with a particular spatial concentration profile that is a function of the rate of *O_2_^.−^* scavenging, to elicit a cell-wide response. A suprathreshold level of cytoplasmic *O_2_^.−^* must reach the neighboring mitochondrion for the response to be regenerative, eliciting the O_2_^.−^-mediated opening of IMAC. In the model, the open probability of IMAC increases as a function of O_2_^.−^, which alters the IMAC conductance versus ΔΨ_m_ relationship (see Eq.50 in the Supplementary Materials). The initial IMAC opening leads to a regenerative increase in ROS because the increased energy dissipation accelerates respiration, in turn increasing the number of electrons shunting to O_2_^.−^, which crosses the inner membrane to further activate IMAC.

**Figure 10:**
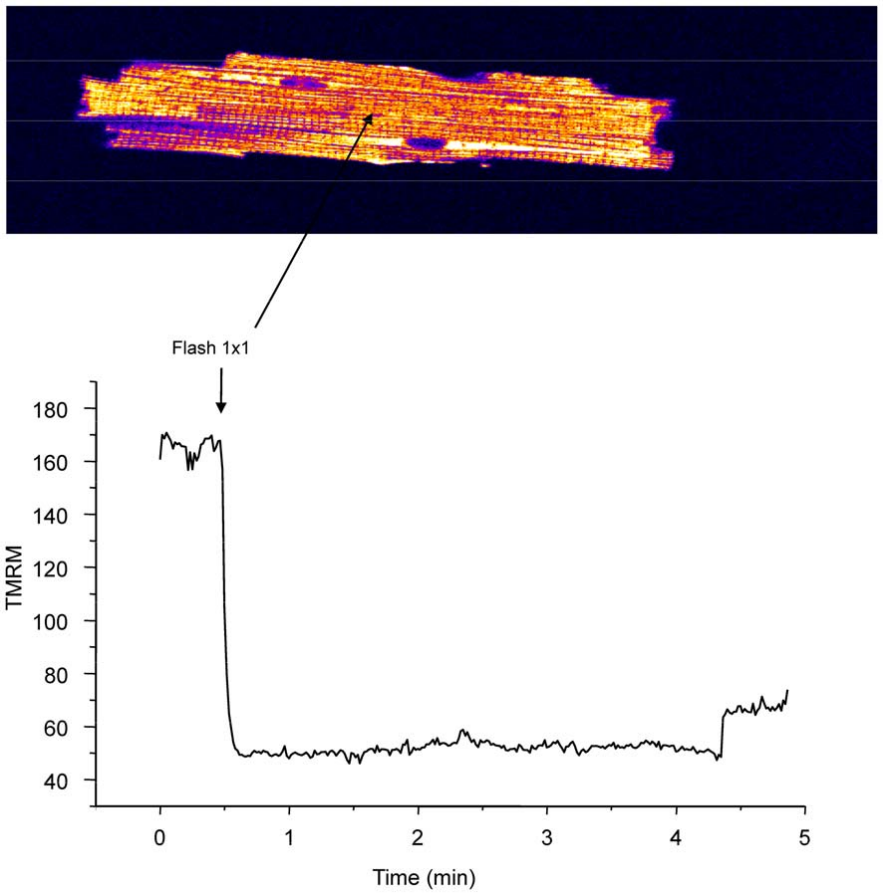
Depolarization of even a single mitochondrion can evoke ΔΨ_m_ collapse of almost the entire network when a cell is at the edge of criticality. Mitochondrial depolarization was triggered with a laser flash (1×1 pixel) in cardiomyocytes labeled with 100 nM TMRE. Frame acquisition was every 500 msec. Other imaging conditions were as described in Materials and Methods.

It is important to note that the global response is very much dependent on both the arrangement of mitochondria in the network and also the number of mitochondria close to the threshold. Before the first ΔΨ_m_ depolarization, the balance of *O_2_^.−^* production to *O_2_^.−^* scavenging must approach the threshold of oxidative stress in approximately 60% of the mitochondria, at which point a small perturbation can cause the synchronous collapse (and/or oscillation) of ΔΨ_m_ in the mitochondrial network. We referred to this vulnerable state as *mitochondrial criticality* [2],[9]. A limitation of this first RD-RIRR model is that it does not entirely reproduce the collective properties of network close to the critical state. For example, when we trigger ΔΨ_m_ oscillations by laser flash, the local increase in ROS load on the network is enough to induce a gradual accumulation of oxidative stress throughout the cell, and the bulk of the network gets closer and closer to criticality. The first global wave of depolarization does not usually happen instantaneously after the trigger, but often occurs after several minutes of delay: it presumably originates from a region having a suprathreshold level of ROS to trigger IMAC opening, and neighbors that are also close to the threshold. The phase of initial spreading of the “oxidative load” after laser stimulation is a collective network property that is difficult to represent with this simple reaction-diffusion model (indeed, modeling the behavior of critical systems is a nascent science). At the edge of criticality, depolarization of even a single mitochondrion can evoke collapse of almost the entire network (Fig. 10, see also Supplemental Materials Video S4). Alternatively, we can induce a critical state of the mitochondrial network by depleting the antioxidant defenses of the cell (globally) with diamide (see [10]). In this condition, we hypothesize that a local increase of ROS above threshold in any part of the 3D network could evoke RIRR. Specifically, the fast synchronized ΔΨ_m_ depolarization-repolarization cycles and waves are the aspects of the phenomenon that are well-reproduced by the 2D RD-RIRR model. Another limitation of current 1D and 2D models is that in the actual experiments, the *O_2_^.−^* can, of course, diffuse both laterally and vertically to other mitochondria, that is, in all 3 dimensions. Presumably, the ROS will have a similar diffusion rate in all directions, although this might depend on the structural organization of the organelles, membranes, and myofilaments. A 3D network would introduce additional triggerable ROS sources above and below the mitochondrion, which could, theoretically, alter the rate of wave propagation. This could be explored in the future by extending the model to three dimensions; however, we do not think that the major conclusions of the study regarding the properties of the excitable system will be substantially different.

The RD-RIRR simulations revealed a novel aspect of RIRR-mediated coupling. A small number of oscillating mitochondria could elicit oscillations in the entire network in both the 1D and 2D models, even though the parameters were set for stable behavior in most of the network (i.e., the percentage of respiration leaking to ROS was below threshold for independent oscillation). This indicates that entrainment by forced oscillation might also occur in the mitochondrial network. In this case, the combined effects of subthreshold ROS release and ROS diffusion from nearby mitochondria exceed the threshold for a regenerative response. This behavior is evident as recruitment of more and more mitochondria into the oscillatory cluster after several cycles of oscillation (as shown in Fig. 8D and the corresponding movie in the Supplemental Materials, Video S3).

While focusing on a specific mechanism of RIRR (i.e., IMAC-mediated), the present findings also provide general theoretical support for mitochondrial communication via RIRR. The RD-RIRR model simulations confirm that *O_2_^.−^* diffusion occurring locally between neighboring mitochondria over a distance of a few microns is sufficient for propagation and synchronization of ΔΨ_m_ depolarization over a larger distance. RIRR involving PTP-dependent depolarization [15],[32] can be readily incorporated into the model in the future; however, at present it is unclear what additional factors besides ROS may be required to evoke this more pronounced, and typically irreversible, cell death event. In our own experiments, we have demonstrated that the IMAC mechanism occurs with mild-to-moderate oxidative stress (by GSH depletion or lower *O_2_^.−^* levels) while PTP activation occurs during more severe stress, e.g. with further GSH depletion [10], or, in the present study, at higher *O_2_^.−^* concentrations. Mitochondrial Ca^2+^ overload is also thought to be a requirement for PTP activation as well [33],[34]. Hence, it is necessary to gain a better understanding of the interplay between the ROS species and the mitochondrial Ca^2+^ load in the activation of the PTP in the intact myocyte before a comprehensive PTP activation model can be constructed.

In summary, we show that the autocatalytic release of *O_2_^.−^* and diffusion between mitochondria are the essential components of propagated RIRR between mitochondria in a closely packed array like that found in the cardiomyocyte. The kinetics and emergent spatiotemporal patterns of ΔΨ_m_ depolarization in the RD-RIRR mitochondrial network are modulated by *O_2_^.−^* production, *O_2_^.−^* scavenging, and diffusion. The highly interdependent nature of the mitochondria as a network of oscillators also suggests that synchronization by forced oscillation may occur when only a part of the network is perturbed.

## Materials and Methods

### 

#### Model development

The mitochondrial reaction-diffusion ROS-induced ROS release (RD-RIRR) model describes a network in which neighboring mitochondria are chemically coupled by *O_2_^.−^*, which increases the open probability of IMAC at an external activation site, as in the single mitochondrial energetics RIRR model (referred to herein as ME-RIRR) [16]. The dynamics of the state variables of each node (i.e., mitochondrion) in the network follow the ME-RIRR, which is comprised of a system of 15 ordinary differential equations describing the TCA cycle, oxidative phosphorylation, mitochondrial Ca^2+^ handling (the Ca^2+^ uniporter and the Na/Ca exchanger), *O_2_^.−^* production, transport and scavenging, and the properties of the IMAC.

#### One dimensional (1D) RD-RIRR model

A 1D version of the RD-RIRR model for up to 50 mitochondria (see figure legends for details) connected end-to-end was constructed for a detailed examination of mitochondrial neighbor-neighbor interactions. Inter-organellar communication is solely via cytoplasmic *O_2_^.−^* (*O_2_^.−^_i_*) diffusion.

The partial differential equation describing the spatiotemporal dynamics of O_2_^.−^_i_ concentration (*C*_O2_^.−^_i_) is as follows:
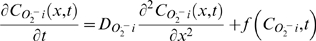
(1)


Boundary conditions: 
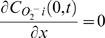
 and 
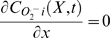


Initial conditions: 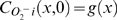


where *D*_O2_^.−^_i_ is the cytoplasmic *O_2_^.−^* diffusion coefficient, X is the total length of the row in the 1D network, and 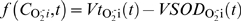. *Vt_O2_^.−^_i_* is the rate of *O_2_^.−^* transport (release) from the mitochondrion (*via* IMAC), and *VSOD_O2_^.−^_i_* is the *O_2_^.−^*_i_ scavenging rate by Cu,Zn superoxide dismutase (SOD). The function g(x) describes the distribution of *O_2_^.−^_i_* at time 0 (the initial condition).

The numerical simulation of the system is performed with a finite difference method, whereby the spatial component at j^th^ node is approximated by the following expression:
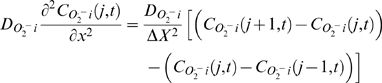
(2)where ΔX is the spacing between two discrete nodes of the network that corresponds to the distance between individual mitochondria. The expressions *Vt_O2_^.−^_i_* and *V_SOD_*, as well as the complete description of the ME-RIRR model, are as previously described [16], as are the model parameters and variable initial values, unless specifically varied in the present study (e.g., the *shunt*, which represents the fraction of respiration diverted to *O_2_^.−^* generation, and *etSOD*, the concentration of Cu, Zn superoxide dismutase) (see also Supplemental Materials, Tables S1, S2, and S3). As mitochondrial membrane potential (Ψ_m_) is affected by inner membrane anion channels (IMAC) (Supplemental Materials, Eq.1), which are activated by elevated surrounding (cytoplasmic) *O_2_^.−^_i_* (table S1.6, E49), the *O_2_^.−^_i_* production and diffusion from one mitochondrion could induce opening of IMAC and Ψ_m_ depolarization in its closest neighbors (through the ROS-induced-ROS-release mechanism).

In order to reduce the computation time, we assume that the cytoplasmic region outside each mitochondrion is homogeneous. We also assume that ΔΨ_m_, ion, and metabolite concentrations are uniform within each mitochondrion. The model was coded in C^++^ and run on a desktop computer. The ordinary differential equations (ODEs) were integrated numerically by CVODE, a stiff ODEs solver that uses variable coefficient Adams and BDF methods (http://citeseer.ist.psu.edu/1230.html). Of note, the C++ code includes an implementation of L'Hopital's rule to take into account an anomaly in the expression for the Ca^2+^ uniporter, which becomes indeterminate at 91 mV. L'Hôpital's rule converts the indeterminate form to a determinate one; however, as the change in ΔΨ_m_ is so rapid, the exception is rarely invoked because that specific point is seldom reached (the integrator typically “steps over” this point).

#### Two dimensional (2D) RD-RIRR model

The thin optical sectioning ability of two-photon laser scanning fluorescence microscopy can be used to examine the behavior of the mitochondrial network in a single plane of a cardiomyocyte [8]. To compare the results of optical imaging experiments in cardiomyocytes subjected to oxidative stress with the computational model, a 2D RD-RIRR model was developed. Each non-boundary node (mitochondrion) in the network was considered to have four nearest neighbors for *O_2_^.−^_i_* interaction (Fig. 1B). At each node (j,k) of the 2D network, *O_2_^.−^_i_* dynamics is described by the mass balance equation based on *O_2_^.^_i_* reaction and diffusion:
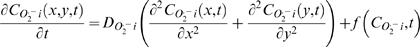
(3)


Boundary conditions: 
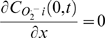
; 
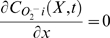


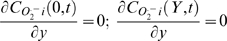



Initial conditions: 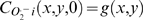


As described above, the spatial coordinates, x and y, are subjected to discretization to numerically solve the system by the finite difference method. The same non-flux boundary conditions are used as in the 1D model. X and Y indicate the total lengths in the dimensions x and y, respectively.

To solve this large nonlinear network consisting of 500 (50×10) mitochondria (each node described by 15 state variables), a high performance parallel computer was used. To be suitable for parallel computation, Eq. (3) was rewritten in the matrix form using forward Euler method to approximate the time derivative of *C*_O2_^.−^_i_ at each node (j,k):
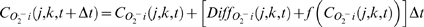
(4)where *C*_O2_^.−^_i_ *(j,k,t)* represents *O_2_^.−^_i_* concentration surrounding the (*j^th^,k^th^*) mitochondrion at instant *t*, *and* Δt is time step size. The matrix *Diff_O2_^.−^_i_(j,k,t)* represents the *O_2_^.−^_i_* diffusion from this node (mitochondrion) to its neighbors (assuming the distance between two neighboring mitochondria are identical in the x and y directions):
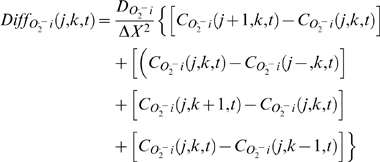
(5)


A data parallel paradigm was used to parallelize this model, where each process simulates only a portion of the global network, and it is responsible for updating its own nodes. This form of parallelization is the most suitable for this problem [35], as it reduces the inter-processes communication to the bare minimum, because only the *C*_O2_^.−^_i_ of the neighboring mitochondria need be communicated. The data (i.e., *C*_O2_^.−^_i_) retrieval between processors was done by MPI (message passing interface) point-to-point communication routines. A mixture of blocking and non-blocking MPI calls (*MPI_Send/MPI_Recv* and *MPI_ISend/MPI_IRecv/MPI_Wait*) was used to avoid deadlocks during data transfer as well as to minimize the cost of communication. As previous experimental and theoretical studies [9] have suggested that locally-restricted *O_2_^.−^_i_* diffusion underlies mitochondrial neighbor-neighbor interaction, especially when one considers the short half-life and scavenging capacity of the cytoplasm, very low values of the diffusion coefficient, *D_O2_^.−^_i_*, were used in the model.

#### Model simulation strategy

Network simulations were used to investigate the potential mechanisms of propagation of mitochondrial RIRR and to examine how synchronization might occur during the self-sustained and highly coordinated cell-wide mitochondrial network oscillations observed experimentally under pathological conditions [8]. In the first simulations, the 1D RD-RIRR network model was used to investigate the interactions between neighboring mitochondria that were within the oscillatory parametric space, i.e., how synchronization of the oscillators was achieved in the network through *O_2_^.−^* diffusion. Specifically, an element in the center of the row was initially set at the completely depolarized state (i.e., low ΔΨ_m_ and high *O_2_^.−^* concentration) while others in the array were set at the polarized states (both groups of mitochondria had identical parameters). The network oscillatory behavior was then simulated with or without *O_2_^.−^* coupling (through diffusion). Next, the effect of oxidative stress on the network responses was simulated by locally increasing the fraction of ROS production (*shunt*) to depolarize ΔΨ_m_ in an element in the center of the row to test whether it can entrain the oscillations of the rest of the mitochondria in the network, which were in the non-oscillatory parametrical domain. Furthermore, the propagation of mitochondrial depolarization waves was simulated using the 2D model. Finally, the effect of diffusion coefficient or ROS scavenging capability on the rate of wave propagation was quantitatively investigated through model simulations.

#### Cardiomyocyte isolation and handling

Cardiomyocytes were prepared from adult guinea-pig hearts by enzymatic digestion and experimental methods were as previously reported [8],[10]. Briefly, after isolation, cells were either immediately used for imaging or stored in Dulbecco's Modification of Eagle's Medium (10-013 DMEM, Mediatech, Inc. Virginia) containing 5% fetal bovine serum and 1% penicillin/streptomycin in a 5% CO_2_ incubator at 37°C for at least 2 hr before assays. Experimental recordings were started after resuspending the cells in a modified Tyrode's solution containing (in mM): 140 NaCl, 5 KCl, 1 MgCl_2_, 10 HEPES, 1 CaCl_2_, pH 7.5 (adjusted with NaOH), supplemented with 10 mM glucose. The perfusion chamber containing the cardiomyocytes was thermostatically controlled at 37°C with unrestricted access to atmospheric oxygen on the stage of a Nikon E600FN upright microscope.

#### Cardiomyocyte permeabilization and loading with fluorescent probes

Myocytes (which were stored in DMEM for 2 hr) were handled and permeabilized as described in Aon et al. [22]. Briefly, the cells were loaded with the fluorescent probes tetramethylrhodamine methyl ester (TMRM, 100 nM) and 5-(–6)-chloromethyl-2′,7′-dichlorohydrofluorescein diacetate (CM-H_2_DCFDA, 2 µM) (Invitrogen-Molecular Probes, Eugene, OR) on the stage of the microscope for at least 20 min at 37°C to monitor ΔΨ_m_ or O_2_^.−^, respectively. After loading, the cells were partially permeabilized by perfusing the chamber for 2.5 min with a solution containing 25_µg/ml saponin, in the presence of a 300∶1 GSH∶GSSG ratio, and (in mM): 130 potassium methanesulfonate, 20 KCl, 0.5 MgCl2, 10 HEPESNa, 0.1 EGTA, 3 ATP, 5 pyruvate; the pH was adjusted to 7.2 with KOH. Potassium superoxide (KO_2_; Alfa Aesar, Johnson Matthey Co.) was used as an exogenous *O_2_^.−^* donor and was applied at concentrations of 10, 20 and 30 µM *via* superfusion of the cell. The Amplex Red assay (Molecular Probes), which detects *O_2_^.−^* indirectly using SOD to convert *O_2_^.−^* to H_2_O_2_, was used to assess the levels of *O_2_^.−^* delivered by KO_2_. Stoichiometric amounts of *O_2_^.−^* were recovered from KO_2_ dissolved in deionized water kept on ice for up to 3 hr.

#### Two-photon microscopy

The equipment and methods utilized for imaging isolated cardiomyocytes were as previously described [8],[16]. Images were recorded using a two-photon laser scanning microscope (Bio-Rad MRC-1024MP) with excitation at 740 nm. The red emission of TMRM was collected at 605±25 nm and the green emission of CM-DCF was recorded at 525±25 nm simultaneously with a frame interval of 3.5 s.

## Supporting Information

Table S1 Mitochondrial energetics and ROS-induced-ROS-release model equations.
(0.13 MB PDF)

Table S2 Mitochondrial energetics and ROS-induced-ROS-release model (ME-RIRR) parameters.
(0.08 MB PDF)

Table S3 Mitochondrial energetics and ROS-induced-ROS-release model (ME-RIRR): states variables initial values (mM).
(0.01 MB PDF)

Video S1 Image sequence illustrating the effect of 10–20µM KO_2_ on ΔΨm (upper) and ROS (lower). Guinea-pig ventricular myocyte was loaded with TMRM and CM-DCF and then partially permeabilized with 25µg/ml saponin as described in methods. Image frames were collected every 3.5 seconds using a two-photon laser scanning fluorescence microscope. Note localized oscillations in mitochondrial ΔΨm.
(2.45 MB AVI)

Video S2 Mitochondrial permeability transition induced by 30µM KO_2_. Prolonged exposure to 30µM KO_2_ induced a mitochondrial permeability transition, as evidenced by loss of both TMRM and CM-DCF (500MW) from the mitochondrial matrix. Images were acquired as described in the material and methods.
(10.42 MB AVI)

Video S3 Image sequence of a pair of TMRM-loaded canine ventricular myocytes (cell-cell junction indicated by the white arrow in left panel) which displayed 22 consecutive oscillations that originated at the end of the righthand cell. Images within each panel were acquired at a 1 second frame rate. Entrainment was indicated by an increase in the area of the oscillating cluster after a number of cycles until the whole cell (up to the border of the neighboring cell) was included.
(11.02 MB AVI)

Video S4 Image sequence of Figure S3 showing that even a single mitochondrion can evoke collapse of almost the entire network when the cell is at the edge of criticality.Mitochondrial depolarization were triggered with a laser flash (1×1 pixel) in cardiomyocytes labeled with 100 nM TMRE. Frame acquisition was every 500 msec. Other imaging conditions were as described in Methods.
(3.42 MB AVI)
