# Supplementary material for: A Reaction-Diffusion Model of ROS-Induced ROS Release in a Mitochondrial Network
Source: PLoS Comput Biol. 2010 Jan 29;6(1):e1000657. doi: 10.1371/journal.pcbi.1000657 (PMC2813265; doi:10.1371/journal.pcbi.1000657)
Supplement: Table S1 — Mitochondrial energetics and ROS-induced-ROS-release model equations. (0.13 MB PDF) [file pcbi.1000657.s001.pdf]

Table S1. Mitochondrial energetics and ROS-induced-ROS-release model equations.

S1.1. Mitochondrial membrane potential ($\Delta\Psi_m$)

$$\frac{d\Delta\Psi_m}{dt} = \frac{V_{He} + V_{He(F)} - V_{Hu} - V_{ANT} - V_{HLeak} - V_{NaCa} - 2V_{uni} - V_{IMAC}}{C_{mito}}$$	E1

S1.2. Energy metabolites mass balance equations

$\frac{d\,[ADP]_m}{dt} = V_{ANT} - V_{ATPase} - V_{SL}$	E2
$[ATP]_m = C_A - [ADP]_m$	E3
$\frac{d[NADH]}{dt} = - V_{O2} + V_{IDH} + V_{KGDH} + V_{MDH}$	E4
$\frac{d[ISOC]}{dt} = V_{ACO} - V_{IDH}$	E5
$\frac{d[\alpha KG]}{dt} = V_{IDH} - V_{KGDH} + V_{AAT}$	E6
$\frac{d[SCoA]}{dt} = V_{KGDH} - V_{SL}$	E7
$\frac{d[Suc]}{dt} = V_{SL} - V_{SDH}$	E8
$\frac{d[FUM]}{dt} = V_{SDH} - V_{FH}$	E9
$\frac{d[MAL]}{dt} = V_{FH} - V_{MDH}$	E10
$\frac{d[OAA]}{dt} = V_{MDH} - V_{CS} - V_{AAT}$	E11
$[CIT] = C_{Kint} - \left(\begin{array}{l} [ISOC] + [\alpha KG] + [SCoA] + [Suc] \\ \qquad + [FUM] + [MAL] + [OAA] \end{array} \right)$	E12
$\frac{d[O2^{\cdot -}]_m}{dt} = shunt\,.\,V_{O_2} - V_{ROS}^{Tr}$	E13
$\frac{d[O2^{\cdot -}]_i}{dt} = V_{ROS}^{Tr} - V_{SOD}$	E14
$\frac{d[H_2O_2]}{dt} = V_{SOD} - V_{CAT} - V_{GPX}$	E15
$\frac{d[GSH]}{dt} = V_{GR} - V_{GPX}$	E16
$\frac{d[Ca^{2+}]_m}{dt} = \delta(V_{uni} - V_{NaCa})$	E51

S1.3. Tricarboxylic acid cycle reaction rates

$$V_{CS} = k_{cat}^{CS}\, E_T^{CS}\left(1 + \frac{K_M^{AcCoA}}{[AcCoA]} + \frac{K_M^{OAA}}{[OAA]} + \frac{K_M^{AcCoA}}{[AcCoA]}\,\frac{K_M^{OAA}}{[OAA]}\right)^{-1} \qquad \text{E17}$$

$$V_{ACO} = k_f^{ACO}\left([CIT] - \frac{[ISOC]}{K_E^{ACO}}\right) \qquad \text{E18}$$

$$f_a^{IDH} = \left[\left(1 + \frac{[ADP]_m}{K_{ADP}^{a}}\right)\left(1 + \frac{[Ca^{2+}]_m}{K_{Ca}^{a}}\right)\right]^{-1} \qquad \text{E19}$$

$$f_i^{IDH} = \left(1 + \frac{[NADH]}{K_{i,NADH}}\right) \qquad \text{E20}$$

$$V_{IDH} = k_{cat}^{IDH}\, E_T^{IDH}\left[\left(1 + \frac{[H^+]}{k_{h,1}} + \frac{k_{h,2}}{[H^+]}\right) + f_i^{IDH}\left(\frac{K_M^{NAD}}{[NAD]}\right) + f_a^{IDH}\left(\frac{K_M^{ISOC}}{[ISOC]}\right)^{ni} + f_a^{IDH}\, f_i^{IDH}\left(\frac{K_M^{ISOC}}{[ISOC]}\right)^{ni}\left(\frac{K_M^{NAD}}{[NAD]}\right)\right]^{-1} \qquad \text{E21}$$

$$f_a^{KGDH} = \left[\left(1 + \frac{[Mg^{2+}]}{K_D^{Mg^{2+}}}\right)\left(1 + \frac{[Ca^{2+}]_m}{K_D^{Ca^{2+}}}\right)\right]^{-1} \qquad \text{E22}$$

$$V_{KGDH} = \frac{k_{cat}^{KGDH}\; E_T^{KGDH}}{1 + f_a^{KGDH}\dfrac{K_M^{\alpha KG}}{[\alpha KG]} + f_a^{KGDH}\left(\dfrac{K_M^{NAD}}{[NAD]}\right)^{n_{aKG}}} \qquad \text{E23}$$

$$V_{SL} = k_f^{SL}\left([SCoA][ADP]_m - \frac{[Suc][ATP]_m[CoA]}{K_E^{SL}}\right) \qquad \text{E24}$$

$$V_{SDH} = \frac{k_{cat}^{SDH}\; E_T^{SDH}}{1 + \left(\dfrac{K_M^{Suc}}{[Suc]}\right)\left(1 + \dfrac{[OAA]}{K_{i,sdh}^{OAA}}\right)\left(1 + \dfrac{[FUM]}{K_i^{FUM}}\right)} \qquad \text{E25}$$

$$V_{FH} = k_f^{FH}\left([FUM] - \frac{[MAL]}{K_E^{FH}}\right) \qquad \text{E26}$$

$$f_{h,a} = \left(1 + \frac{[H^+]}{k_{h1}} + \frac{[H^+]^2}{k_{h1}\, k_{h2}}\right)^{-1} + k_{offset} \qquad \text{E27}$$

$$f_{h,i} = \left(1 + \frac{k_{h3}}{[H^+]} + \frac{k_{h3}\ k_{h4}}{[H^+]^2}\right)^{-2} \quad \text{E28}$$

$$V_{MDH} = \frac{k_{cat}^{MDH}\ E_T^{MDH}\ f_{h,a}\ f_{h,i}}{1 + \frac{K_M^{MAL}}{[MAL]}\left(1 + \frac{[OAA]}{K_i^{OAA}}\right) + \frac{K_M^{NAD}}{[NAD]} + \frac{K_M^{MAL}}{[MAL]}\ \left(1 + \frac{[OAA]}{K_i^{OAA}}\right)\frac{K_M^{NAD}}{[NAD]}} \quad \text{E29}$$

$$V_{AAT} = k_f^{AAT}[OAA][GLU]\ \frac{k_{ASP}\ K_E^{AAT}}{\left(k_{ASP}\ K_E^{AAT} + [\alpha KG]\ k_f^{AAT}\right)} \quad \text{E30}$$

S1.4. Oxidative phosphorylation reaction rates

$$V_{O_2} = 0.5\,\rho^{res}\,\frac{\left(r_a + r_{c1}\,e^{\left(\frac{6F\,\Delta\Psi_B}{R\,T}\right)}\right)e^{\left(\frac{A_{res}\,F}{R\,T}\right)} - r_a\,e^{\left(\frac{g\,6\,F\,\Delta\mu_H}{R\,T}\right)} + r_{c2}\,e^{\left(\frac{A_{res}\,F}{R\,T}\right)}\,e^{\left(\frac{g\,6\,F\,\Delta\mu_H}{R\,T}\right)}}{\left(1 + r_1\,e^{\left(\frac{F\,A_{res}}{R\,T}\right)}\right)e^{\left(\frac{6\,F\Delta\Psi_B}{R\,T}\right)} + \left(r_2 + r_3\,e^{\left(\frac{F\,A_{res}}{R\,T}\right)}\right)e^{\left(\frac{g\,6\,F\,\Delta\mu_H}{R\,T}\right)}} \quad \text{E31}$$

$$V_{He} = 6\,\rho^{res}\,\frac{\left(r_a\,e^{\left(\frac{A_{res}\,F}{R\,T}\right)} - (r_a + r_b)\,e^{\left(\frac{g\,6F\,\Delta\mu_H}{R\,T}\right)}\right)}{\left(1 + r_1\,e^{\left(\frac{F\,A_{res}}{R\,T}\right)}\right)e^{\left(\frac{6\,F\Delta\Psi_B}{R\,T}\right)} + \left(r_2 + r_3\,e^{\left(\frac{F\,A_{res}}{R\,T}\right)}\right)e^{\left(\frac{g\,6\,F\,\Delta\mu_H}{R\,T}\right)}} \quad \text{E32}$$

$$A_{res} = \frac{R\,T}{F}\ln\left(K_{res}\sqrt{\frac{[NADH]}{[NAD^+]}}\right) \quad \text{E33}$$

$$[NAD^+] = C_{PN} - [\,NADH] \quad \text{E34}$$

$$V_{He(F)} = \frac{4\,\rho^{res(F)} \times \left(r_a\,e^{\left(\frac{A_{res(F)}\,F}{R\,T}\right)} - (r_a + r_b)\,e^{\left(\frac{g\,6F\,\Delta\mu_H}{R\,T}\right)}\right)}{\left(1 + r_1\,e^{\left(\frac{F\,A_{res(F)}}{R\,T}\right)}\right)e^{\left(\frac{6\,F\Delta\Psi_B}{R\,T}\right)} + \left(r_2 + r_3\,e^{\left(\frac{F\,A_{res(F)}}{R\,T}\right)}\right)e^{\left(\frac{g\,6\,F\,\Delta\mu_H}{R\,T}\right)}} \quad \text{E35}$$

$$A_{res(F)} = \frac{R\,T}{F}\ln\left(K_{res(F)}\sqrt{\frac{[FADH_2]}{[FAD]}}\right) \quad \text{E36}$$

$$V_{ATPase} = -\rho^{F1}\,\frac{\left(10^2\,p_a + p_{c1}\,e^{\left(\frac{3\,F\Delta\Psi_B}{R\,T}\right)}\right)e^{\left(\frac{A_{F1}\,F}{R\,T}\right)} - \left(p_a\,e^{\left(\frac{3\,F\Delta\mu_H}{R\,T}\right)} + p_{c2}\,e^{\left(\frac{A_{F1}\,F}{R\,T}\right)}\,e^{\left(\frac{3\,F\Delta\mu_H}{R\,T}\right)}\right)}{\left(1 + p_1\,e^{\left(\frac{F\,A_{F1}}{R\,T}\right)}\right)e^{\left(\frac{3\,F\Delta\Psi_B}{R\,T}\right)} + \left(p_2 + p_3\,e^{\left(\frac{F\,A_{F1}}{R\,T}\right)}\right)e^{\left(\frac{3\,F\Delta\mu_H}{R\,T}\right)}} \quad \text{E37}$$

$$V_{Hu} = -3\,\rho^{F1}\,\frac{10^2\,p_a\left(1 + e^{\left(\frac{F\,A_{F1}}{R\,T}\right)}\right) - (p_a + p_b)\,e^{\left(\frac{3\,F\,\Delta\mu_H}{R\,T}\right)}}{\left(1 + p_1\,e^{\left(\frac{F\,A_{F1}}{R\,T}\right)}\right)e^{\left(\frac{3\,F\Delta\Psi_B}{R\,T}\right)} + \left(p_2 + p_3\,e^{\left(\frac{F\,A_{F1}}{R\,T}\right)}\right)e^{\left(\frac{3\,F\,\Delta\mu_H}{R\,T}\right)}} \quad \text{E38}$$

$A_{F1} = \frac{\mathrm{R\ T}}{\mathrm{F}} \ln\left(\mathrm{K}_{\mathrm{F1}} \frac{[\mathrm{ATP}]_{\mathrm{m}}}{[ADP]_m\ \mathrm{Pi}} \right)$	E39
$\mathrm{V}_{\mathrm{Hleak}} = \mathrm{g}_{\mathrm{H}}\ \Delta\mu_{\mathrm{H}}$	E40
$\Delta\mu_{\mathrm{H}} = -2.303 \frac{\mathrm{R\ T}}{\mathrm{F}} \Delta\mathrm{pH} + \Delta\Psi_{\mathrm{m}}$	E41

S1.5. Mitochondrial Ca^{2+} uniporter and and Ca^{2+}-Na^{+} exchanger rates

$$V_{uni} = V_{max}^{uni} \frac{\frac{[Ca^{2+}]_i}{K_{trans}}\left(1 + \frac{[Ca^{2+}]_i}{K_{trans}}\right)^3 \frac{2\,F\,\left(\Delta\Psi_m - \Delta\Psi^o\right)}{R\;T}}{\left(\left(1 + \frac{[Ca^{2+}]_i}{K_{trans}}\right)^4 + \frac{L}{\left(1 + \frac{[Ca^{2+}]_i}{K_{act}}\right)^{n_a}}\right)\left(1 - e^{\left\{\frac{-2\,F\,\left(\Delta\Psi_m - \Delta\Psi^o\right)}{R\;T}\right\}}\right)} \quad \text{E42}$$

$$V_{NaCa} = V_{max}^{NaCa} \frac{e^{\left(\frac{b\,F\left(\Delta\Psi_m - \Delta\Psi^\circ\right)}{R\,T}\right)} e^{\left(\ln\frac{[Ca^{2+}]_m}{[Ca^{2+}]_i}\right)}}{\left(1+\frac{K_{Na}}{[Na^{+}]_i}\right)^n \left(1+\frac{K_{Ca}}{[Ca^{2+}]_m}\right)} \quad \text{E43}$$

S1.6. ROS-induced-ROS-release rates

$$V_{SOD} = \frac{2\,k^1_{SOD}\,k^5_{SOD}\left(k^1_{SOD} + k^3_{SOD}\left(1+\frac{[H_2O_2]}{K_i^{H2O2}}\right)\right)E^T_{SOD}\,[O_2^{\cdot -}]}{k^5_{SOD}\left(2\,k^1_{SOD} + k^3_{SOD}\left(1+\frac{[H_2O_2]}{K_i^{H2O2}}\right)\right) + [O_2^{\cdot -}]\,k^1_{SOD}\,k^3_{SOD}\left(1+\frac{[H_2O_2]}{K_i^{H2O2}}\right)} \quad \text{E44}$$

$$V_{CAT} = 2\;k^1_{CAT}\;E^T_{CAT}\,[H_2O_2]\,e^{-fr\,[H_2O_2]} \quad \text{E45}$$

$$V_{GPX} = \frac{E^T_{GPX}\,[H_2O_2][GSH]}{\Phi_1\,[GSH] + \Phi_2\,[H_2O_2]} \quad \text{E46}$$

$$V_{GR} = \frac{k^1_{GR}\;E^T_{GR}}{1 + \frac{K_M^{GSSG}}{[GSSG]} + \frac{K_M^{NADPH}}{[NADPH]} + \frac{K_M^{GSSG}}{[GSSG]}\frac{K_M^{NADPH}}{[NADPH]}} \quad \text{E47}$$

$$V^{Tr}_{ROS} = j\,.\frac{V_{IMAC}}{\Delta\Psi_m}\left(\Delta\Psi_m - \tfrac{RT}{F}\log\left(\frac{[O_2^{\cdot -}]_m}{[O_2^{\cdot -}]_i}\right)\right) \quad \text{E48}$$

$$V_{IMAC} = \left(a + \frac{b}{1+\frac{K_{cc}}{[O_2^{\cdot -}]_i}}\right)\left(G_L + \frac{G_{max}}{1+e^{\left(\kappa\left(\Delta\Psi^b_m - \Delta\Psi_m\right)\right)}}\right)\Delta\Psi_m \quad \text{E49}$$

$$G_T = [GSH] + 2\times[GSSG] \quad \text{E50}$$
