# Supplementary material for: A Reaction-Diffusion Model of ROS-Induced ROS Release in a Mitochondrial Network
Source: PLoS Comput Biol. 2010 Jan 29;6(1):e1000657. doi: 10.1371/journal.pcbi.1000657 (PMC2813265; doi:10.1371/journal.pcbi.1000657)
Supplement: Table S2 — Mitochondrial energetics and ROS-induced-ROS-release model (ME-RIRR) parameters. (0.08 MB PDF) [file pcbi.1000657.s002.pdf]

Table S2. Mitochondrial energetics and ROS-induced-ROS-release model (ME-RIRR) parameters.

S2.1 Tricarboxylic acid cycle

Symbol	Value	Units	Description	Eq.
[AcCoA]	1.0	mM	Acetyl CoA concentration	E17
k_{cat}^{CS}	0.05	ms^{-1}	Catalytic constant of CS	E17
E_T^{CS}	0.4	mM	Concentration of CS	E17
K_M^{AcCoA}	1.26×10^{-2}	mM	Michaelis constant for AcCoA	E17
K_M^{OAA}	6.4×10^{-4}	mM	Michaelis constant for OAA	E17
C_{Kint}	1.0	mM	Sum of TCA cycle intermediates' concentration	E12
k_f^{ACO}	1.25×10^{-2}	ms^{-1}	Forward rate constant of ACO	E18
K_E^{ACO}	2.22		Equilibrium constant of ACO	E18
K_{ADP}^{a}	0.62	mM	Activation constant by ADP	E19
K_{Ca}^{a}	0.0005	mM	Activation constant for Ca^{2+}	E19
$K_{i,NADH}$	0.19	mM	Inhibition constant by NADH	E20
k_{cat}^{IDH}	0.03	ms^{-1}	Rate constant of IDH	E21
E_T^{IDH}	0.109	mM	Concentration of IDH	E21
$[H^+]$	2.5×10^{-5}	mM	Matrix proton concentration	E21
$k_{h,1}$	8.1×10^{-5}	mM	Ionization constant of IDH	E21
$k_{h,2}$	5.98×10^{-5}	mM	Ionization constant of IDH	E21

K_M^{ISOC}	1.52	mM	Michaelis constant for isocitrate	E21
ni	2.0		Cooperativity for isocitrate	E21
K_M^{NAD}	0.923	mM	Michaelis constant for NAD^+	E21
$K_D^{Mg^{2+}}$	0.0308	mM	Activation constant for Mg^{2+}	E22
$K_D^{Ca^{2+}}$	1.27×10^{-3}	mM	Activation constant for Ca^{2+}	E22
E_T^{KGDH}	0.5	mM	Concentration of KGDH	E23
k_{cat}^{KGDH}	0.05	ms^{-1}	Rate constant of KGDH	E23
$K_M^{\alpha KG}$	1.94	mM	Michaelis constant for αKG	E23
K_M^{NAD}	38.7	mM	Michaelis constant for NAD	E23
$n_{\alpha KG}$	1.2		Hill coefficient of KGDH for αKG	E23
Mg^{2+}	0.4	mM	Mg^{2+} concentration in mitochondria	E22
k_f^{SL}	5.0×10^{-4}	mM^{-1} ms^{-1}	Forward rate constant of SL	E24
K_E^{SL}	3.115		Equilibrium constant of the SL reaction	E24
[CoA]	0.02	mM	Coenzyme A concentration	E24
k_{cat}^{SDH}	3.0×10^{-3}	ms^{-1}	Rate constant of SDH	E25
E_T^{SDH}	0.5	mM	SDH enzyme concentration	E25
K_M^{Suc}	0.03	mM	Michaelis constant for succinate	E25
K_i^{FUM}	1.3	mM	Inhibition constant by fumarate	E25
$K_{i,sdh}^{OAA}$	0.15	mM	Inhibition constant by oxalacetate	E25

k_f^{FH}	3.32×10^{-3}	ms^{-1}	Forward rate constant for FH	E26
K_E^{FH}	1.0		Equilibrium constant of FH	E26
k_{h1}	1.13×10^{-5}	mM	Ionization constant of MDH	E27
k_{h2}	26.7	mM	Ionization constant of MDH	A153
k_{h3}	6.68×10^{-9}	mM	Ionization constant of MDH	E28
k_{h4}	5.62×10^{-6}	mM	Ionization constant of MDH	E28
k_{offset}	3.99×10^{-2}		pH-independent term in the pH activation factor of MDH	E27
k_{cat}^{MDH}	0.111	ms^{-1}	Rate constant of MDH	E29
E_T^{MDH}	0.154	mM	Total MDH enzyme concentration	E29
K_M^{MAL}	1.493	mM	Michaelis constant for malate	E29
K_i^{OAA}	3.1×10^{-3}	mM	Inhibition constant for oxalacetate	E29
K_M^{NAD}	0.2244	mM	Michaelis constant for NAD^+	E29
[GLU]	10.0	mM	Glutamate concentration	E30
k_f^{AAT}	6.44×10^{-4}	ms^{-1}	Forward rate constant of AAT	E30
K_E^{AAT}	6.6		Equilibrium constant of AAT	E30
k_{ASP}	1.5×10^{-6}	ms^{-1}	Rate constant of aspartate consumption	E30

S2.2. Oxidative phosphorylation

Symbol	Value	Units	Description	Eq.
r_a	6.394×10^{-13}	ms^{-1}	Sum of products of rate constants	E31
r_b	1.762×10^{-16}	ms^{-1}	Sum of products of rate constants	E32
r_{c1}	2.656×10^{-22}	ms^{-1}	Sum of products of rate constants	E31
r_{c2}	8.632×10^{-30}	ms^{-1}	Sum of products of rate constants	E31
r_1	2.077×10^{-18}		Sum of products of rate constants	E31
r_2	1.728×10^{-9}		Sum of products of rate constants	E31
r_3	1.059×10^{-26}		Sum of products of rate constants	E31
ρ^{res}	3.0×10^{-3}	mM	Concentration of electron carriers (respiratory complexes I-III-IV)	E31
K_{res}	1.35×10^{18}		Equilibrium constant of respiration	E33
$\rho^{res(F)}$	3.75×10^{-4}	mM	Concentration of electron carriers (respiratory complexes II-III-IV)	E35
$\Delta\Psi_B$	50	mV	Phase boundary potential	E31
g	0.85		Correction factor for voltage	E31
$K_{res(F)}$	5.765×10^{13}		Equilibrium constant of $FADH_2$ oxidation	E36
[FADH2]	1.24	mM	Concentration of $FADH_2$ (reduced)	E36
[FAD]	0.01	mM	Concentration of FAD (oxidized)	E36
p_a	1.656×10^{-8}	ms^{-1}	Sum of products of rate constants	E37
p_b	3.373×10^{-10}	ms^{-1}	Sum of products of rate constants	E38
p_{c1}	9.651×10^{-17}	ms^{-1}	Sum of products of rate constants	E37
p_{c2}	4.585×10^{-17}	ms^{-1}	Sum of products of rate constants	E37

p_1	1.346×10^{-8}		Sum of products of rate constants	E37
p_2	7.739×10^{-7}		Sum of products of rate constants	E37
p_3	6.65×10^{-15}		Sum of products of rate constants	E37
ρ^{F1}	1.5	mM	Concentration of F_1F_0-ATPase	E37
K_{F1}	1.71×10^{6}		Equilibrium constant of ATP hydrolysis	E37
Pi	2.0	mM	Inorganic phosphate concentration	E37
C_A	1.5	mM	Total sum of mito adenine nucleotides	E3
g_H	1.0×10^{-8}	mM ms^{-1} mV^{-1}	Ionic conductance of the inner membrane	E40
ΔpH	-0.6	pH units	pH gradient across the inner memb.	E40
C_{PN}	10.0	mM	Total sum of mito pyridine nucleotides	E34
C_{mito}	1.812×10^{-3}	mM mV^{-1}	Inner membrane capacitance	E1

S2.3 Mitochondrial Ca^{2+} uniporter and Ca^{2+}-Na^+ exchanger

Symbol	Value	Units	Description	Eq.
V_{max}^{uni}	0.0275	mM ms^{-1}	Vmax uniport Ca^{2+} transport	E42
$\Delta\Psi°$	91	mV	Offset membrane potential	E42
K_{act}	3.8×10^{-4}	mM	Activation constant	E42
K_{trans}	0.019	mM	K_d for translocated Ca^{2+}	E42
L	110.0		Keq for conformational transitions in uniporter	E42
n_a	2.8		Uniporter activation cooperativity	E42
V_{max}^{NaCa}	1.0×10^{-4}	mM ms^{-1}	Vmax of Na^+/Ca^{2+} antiporter	E43
b	0.5		$\Delta\Psi_m$ dependence of Na^+/Ca^{2+} antiporter	E43
K_{Na}	9.4	mM	Antiporter Na^+ constant	E43
K_{Ca}	3.75×10^{-4}	mM	Antiporter Ca^{2+} constant	E43
n	3		Na^+/Ca^{2+} antiporter cooperativity	E43
δ	$3.0\ 10^{-4}$		Fraction of free $[Ca^{2+}]_m$	E51

S2.4 ROS induced ROS release

Symbol	Value	Units	Description	Eqn.
G_L	0.0782	mM s^{-1} V^{-1}	Leak conductance for IMAC	E49
G_{max}	7.82	mM s^{-1} V^{-1}	Integral Conductance of IMAC at saturation	E49
a	1.0 10^{-3}		Basal IMAC conductance	E49
b	1.0 10^{4}		Activation factor by cytoplasmic $O_2^{\cdot-}$	E49
κ	70	V^{-1}	Steepness factor	E49
$\Delta\Psi_m^b$	0.004	V	Potential at half saturation	E49
K_{cc}	0.01	mM	Activation constant of IMAC by $O_2^{\cdot-}$	E49
k_{SOD}^1	2.4 10^{6}	mM^{-1} s^{-1}	Second order rate constant of conversion between native oxidized and reduced superoxide dismutase (SOD)	E44
k_{SOD}^3	1.7 10^{4}	mM^{-1} s^{-1}	Second order rate constant of conversion between native reduced SOD and its inactive form	E44
k_{SOD}^5	5.0 10^{-1}	s^{-1}	First order rate constant for conversion between inactive and active oxidized SOD	E44
E_{SOD}^T	0.4 - 2.5 10^{-3}	mM	Intracellular concentration of SOD	E44
K_i^{H2O2}	0.5	mM	Inhibition constant for H_2O_2	E44
k_{CAT}^1	4.8 10^{4}	mM^{-1} s^{-1}	Rate constant of catalase (CAT)	E45
E_{CAT}^T	0.001	mM	Intracellular concentration of CAT	E45
fr	50		Hydrogen peroxide inhibition factor of CAT	E45
E_{GPX}^T	0.00141	mM	Intracellular concentration of glutathione peroxidase (GPX)	E45
Φ_1	0.15-5.0	mM s	Constant for GPX activity	E46
Φ_2	0.5	mM s	Constant for GPX activity	E46
K_M^{GSSG}	1.94	mM	Michaelis constant for oxidized glutathione of glutathione reductase (GR)	E46

K_M^{NADPH}	38.7	mM	Michaelis constant for NADPH of GR	E47
k_{GR}^1	0.0308	s^{-1}	Rate constant of GR	E47
E_{GR}^T	$1.27\ 10^{-3}$	mM	Intracellular concentration of GR	E47
G_T	1 - 2	mM	Total intracellular pool of glutathione	E50
shunt	0.02		Percentage of respiration diverted to ROS production	E13
j	0.12		Fraction of IMAC conductance	E48
