# Supplementary material for: A Reaction-Diffusion Model of ROS-Induced ROS Release in a Mitochondrial Network
Source: PLoS Comput Biol. 2010 Jan 29;6(1):e1000657. doi: 10.1371/journal.pcbi.1000657 (PMC2813265; doi:10.1371/journal.pcbi.1000657)
Supplement: Table S3 — Mitochondrial energetics and ROS-induced-ROS-release model (ME-RIRR): states variables initial values (mM). (0.01 MB PDF) [file pcbi.1000657.s003.pdf]

Table S3. Mitochondrial energetics and ROS-induced-ROS-release model (ME-RIRR): states variables initial values (mM).

Symbol	Description	Value
$[ADP]_m$	Mitochondrial ADP concentration	0.0097
[NADH]	Mitochondrial NADH concentration	2.084
$\Delta\Psi_m$	Inner mitochondrial membrane potential	- 124.7
[ISOC]	Isocitrate concentration (mitochondrial)	0.108
$[\alpha KG]$	α ketoglutarate concentration (mitochondrial)	2.596×10^{-4}
[SCoA]	Succinyl CoA concentration (mitochondrial)	0.89
[Suc]	Succinate concentration (mitochondrial)	4.88×10^{-4}
[FUM]	Fumarate concentration (mitochondrial)	0.0103
[MAL]	Malate concentration (mitochondrial)	0.0038
[OAA]	Oxalacetate concentration (mitochondrial)	4.743×10^{-8}
$[O_2^{\cdot-}]_i$	$O_2^{\cdot-}$ concentration (cytoplasmic)	1.192×10^{-8}
$[O_2^{\cdot-}]_m$	$O_2^{\cdot-}$ concentration (mitocondrial)	0.0238
$[H_2O_2]$	Hydrogen peroxdize (cytoplasmic)	8.821×10^{-9}
[GSH]	Reduced glutathione (cytoplasmic)	0.841
$[Ca^{2+}]_m$	Calcium concentration (mitocondrial)	1.132×10^{-4}
